# Systematic review of economic evaluations on stereotactic ablative radiotherapy (SABR) compared to other radiotherapy techniques or surgical procedures for early-stage non-small cell lung cancer

**DOI:** 10.1186/s12962-023-00415-1

**Published:** 2023-01-16

**Authors:** Fernando Henrique de Albuquerque Maia, Luciana Martins Rozman, Heloisa de Andrade Carvalho, Patrícia Coelho de Soárez

**Affiliations:** 1 Departamento de Medicina Preventiva, Faculdade de Medicina FMUSP, Universidade de Sao Paulo, Av Dr Arnaldo 455, Sao Paulo, SP CEP: 01246903 Brazil; 2 Departamento de Radiologia E Oncologia, Divisao de Radioterapia, Faculdade de Medicina FMUSP, Universidade de Sao Paulo, Sao Paulo, SP Brazil; 3 National Institute of Science and Technology for Health Technology Assessment (IATS), CNPq/Brazil, Brasília, Brazil

**Keywords:** Non-small Cell Lung Cancer (NSCLC), Stereotactic Ablative Radiotherapy (SABR), Cost-Effectiveness Analysis

## Abstract

**Background:**

Stereotactic ablative radiotherapy (SABR) is recommended as first-choice treatment to inoperable early-stage non-small cell lung cancer (NSCLC). However, it is not widely adopted in developing countries, and its cost-effectiveness is unclear. We aimed to perform a systematic review of full economic evaluations (EE) that compared SABR with other radiotherapy or surgical procedures to assess the results and methodological approach.

**Methods:**

The protocol was registered on PROSPERO (CRD42021241640). We included full EE studies with early-stage NSCLC in which one group was submitted to SABR. Studies that were partial EE, included advanced NSCLC or other neoplasm were excluded. We performed the last search on June 2021 in Medline, EMBASE and other databases. The reporting quality were assessed by CHEERS checklist. The main characteristics of each study were tabulated, and the results were presented by a narrative synthesis.

**Results:**

We included nine studies. Three compared radiotherapy techniques, in which SABR was found to be dominant or cost-effective. Six compared SABR with surgery, and in this group, there was not a unanimous decision. All included only direct healthcare costs but varied about categories included. The parameters used in the model-based studies were highly heterogeneous using mixed data from various sources. The items properly reported varied from 29 to 67%.

**Conclusions:**

The studies were all from developed countries and lacked in reporting quality. We recommend that developing countries produce their own studies. More strict alignment to reporting guidelines and use of robust evidence as model parameters are also advised.

**Supplementary Information:**

The online version contains supplementary material available at 10.1186/s12962-023-00415-1.

## Background

Cancer is a significant cause of morbidity and mortality globally, with an estimated 19.3 million new cases and 10 million deaths in 2020. Lung cancer is second among new neoplasms cases, accounting for 11.4% of the total, and the first cause of cancer deaths, imputed for 18.0% of the total [1].

The main risk factor for non-small cell lung cancer (NSCLC) is smoking. Even after smoking cessation, an increased risk for lung cancer remains for many years [2, 3]. This kind of cancer usually has a late diagnosis [4], but this scenario may change with new population screening strategies, such as those recommended by the United States Preventive Services Task Force [5].

The gold standard treatment for early-stage NSCLC is lobectomy [6]. However, the presence of comorbidities can reach 76% of patients [7], which impacts the setting of oncological treatment. Lung diseases, with less than 40% forced expiratory volume in the first second (FEV1), heart diseases, and advanced diabetes mellitus with damage to target organs count among the conditions that may contraindicate surgery [8].

Inoperable patients or those who refuse surgery can reach 18% of the total, and in the absence of any treatment, the median survival is only 9 months [9]. Radiotherapy techniques are generally used in these patients, either conventional radiotherapy (CRT) or stereotactic ablative radiotherapy (SABR).

In CRT, the patient is submitted to a total dose between 60 and 66 Gy, administered in 30 to 35 sessions [10], while SABR applies high doses of radiation precisely directed to the topography of the tumor, issued in 1 to 5 sessions [11]. Although SABR is also known as stereotactic body radiation therapy (SBRT), we will use only SABR to refer to it in this review.

A systematic review recently published approached the comparison of these two techniques in the treatment of stage I NSCLC [12]. Compared to CRT, SABR led to superior survival, with a progression-free survival hazard ratio (HR) of 0.34 [95% confidence interval (CI): 0.25–0.48, P < 0.00001] and an overall survival HR of 0.66 [95% CI: 0.62–0.70, P < 0.00001]. However, from 17 studies included in the meta-analysis, only 2 were randomized controlled trials, and most patients were from 12 retrospective cohorts with heterogeneous treatment regimens.

SABR is recommended by scientific societies as the first-choice treatment for early-stage NSCLC in inoperable patients or those who refuse surgery [13, 14]. Although it is used in the public health system in developed countries [15, 16], it is not widely adopted in developing countries, and its efficiency in these scenarios is unclear.

Adopting a new treatment, particularly at scarce resource scenarios, should rely on economic evaluations (EE) by modeling techniques or assessing real-world data based on patient registries. These studies can estimate costs and health outcomes related to different treatments and then provide information about the efficiency of some therapies compared to other alternatives.

EEs cannot apply to recommend the adoption of therapies in countries other than the one in which it was conducted without assessing the transferability of the study [17]. A detailed description, including the methodologic approach, the assumptions made, and the environment where it took place, shall be raised. Several aspects might limit the transferability, such as the prevalence of the disease and its risk factors, healthcare system organization, provider payment model, clinical guidelines in use, etc.

Systematic reviews of EEs can help policymakers make decisions, as they synthesize all the available evidence and perform critical analysis of it. Moreover, they can also assist authors in designing new studies by stressing methodological issues and their strengths and weaknesses [18]. Therefore, the objective of this review was to carry out a systematic review of full economic evaluations that compared SABR with other radiotherapy or surgical procedures in patients with early-stage NSCLC to review critical methodological issues and inform the design of future EE studies, especially in the context of a developing country.

## Methods

This systematic review was developed according to the methodology presented in a series of articles published in 2016 entitled “How to prepare a systematic review of economic evaluations for informing evidence-based healthcare decisions” [19–21]. The study protocol was registered on PROSPERO (CRD42021241640), and it will be presented accordingly to the Preferred Reporting Items for Systematic Reviews and Meta-Analyzes (PRISMA) [22].

### Composition of the team of reviewers

The team of reviewers included professionals with experience in methodologies for health economic evaluation and radiotherapy experts.

### Data sources and search strategy

We searched the documents in the following electronic databases: MEDLINE via PubMed, Scopus, EMBASE, Virtual Health Library, Cochrane Library / CENTRAL, LILACS, CINAHL Scopus, Web of Science, CRD—NHS Economic Evaluation Database, CRD—Health Technology Assessment Database, International HTA Database, Cost-Effectiveness Analysis Registry, Regional Health Technology Assessment Reports of the Americas (BRISA), EconStor, EconPapers, EconLit Database, The National Institute for Health and Care Excellence (NICE) Evidence Search (Economic Evaluations and HTA), Canadian Agency for Drugs and Technologies in Health (CADHT) and National Commission for the Incorporation of Technologies in SUS (CONITEC). The initial search was performed on October 20, 2020. It was updated on June 3, 2021.

We built the search strategy from a PICOS framework, according to the inclusion and exclusion criteria displayed in Table 1. We adopted controlled vocabulary, corresponding to the subject descriptor officially registered in each database (MeSH term for PubMed and CENTRAL, and EMTREE in EMBASE), considering synonyms, acronyms, and spellings commonly used. No restrictions, such as the date, language, or publication status, were imposed for the initial search. The entire search strategy is available (Additional file 1).

**Table 1:** Components of the research question on the PICOS framework

	Inclusion criteria	Exclusion criteria
Population (P)	Patients with early-stage non-small cell lung cancer	Patients with other primary cancers Patients with advanced lung cancer or metastasis
Intervention (I)	Stereotactic ablative radiotherapy (SABR)	Concomitant use of chemotherapy or immunotherapy
Comparator (C)	Other radiotherapy techniques or surgical treatments	
Outcomes (O)	Incremental cost–effectiveness ratio (ICER), cost–benefit ratio, net benefit, other summary measures	
Design of Study (S)	Full economic evaluation (Cost-minimization analysis, Cost–effectiveness analysis, Cost-utility analysis, or Cost–benefit analysis)	Partial economic evaluations

### Reference management

We compiled the search results in a collection in “Mendeley” software. After removing duplicates, these records were exported to Rayyan online systematic review management software [23].

### Selection

Two reviewers (FHAM and LMR) independently performed the screening of the identified records in Rayyan. In this phase, studies were excluded if they did not answer the research question after reading their title and abstract.

In case of doubt, or if at least one reviewer considered the study eligible, the whole report was sought for retrieval. The full-text reports were submitted to eligibility assessment, and at this phase, an “Excel” sheet was used, with standardized terminology to identify the reasons for exclusion. The spreadsheets filled by each reviewer were compared, and, in cases of discrepancy, a discussion was held to obtain consensus.

### Data extraction

A standardized instrument was built for data extraction from eligible reports. We tested this instrument with two reports to assess its compliance and align terminologies. After validating the instrument, the two reviewers performed the data extraction independently.

The information collected included setting and population, type of study, study perspective, methodology, model parameters, assumptions applied, costs and their composition, results obtained, and principal conclusions.

### Critical appraisal of reports

The eligible studies were also appraised by the two reviewers independently. To assess the quality of the evidence used as parameters in the model-based studies, we used the approach proposed by Cooper et al. [24]. In this analysis, the data components are categorized into five categories: “Clinical effect sizes, adverse events & complications”, “Baseline clinical data”, “Resource use”, “Costs”, and “Utilities”. At each one, the evidence used is assigned with values from 1 + (best evidence) to 6 (weakest evidence). If it was not possible to assess the quality of the evidence, it was set as “Unclear.” If the source did not apply in this classification, it was posted as “Not applicable.”

The reporting quality was assessed by the checklist proposed by the Consolidated Health Economic Evaluation Reporting Standards Statement (CHEERS) [25]. Each item of the checklist was assigned with “Yes,” “Partially,” and “No” or “Not applicable (NA)” according to meeting the criterion.

To assess conflicts of interest, we considered whether any of the authors were affiliated with the manufacturer, whether the manufacturer directly financed the study, and whether the authors reported manufacturer-related competing interests, according to Valachis et al. [26] If any of these items were assigned with “Yes,” we considered that the study presented a potential conflict of interest.

### Summary of the data

We did not perform a meta-analysis because economic evaluation studies are generally heterogeneous and do not have sufficient detail to adjust the results [27]. A narrative synthesis of the studies was carried out from the consolidated data, which adopted rigorous and transparent qualitative techniques to analyze the included studies and the relationships between them, assessing the quality and robustness of the evidence [28].

A qualitative description of the differences between the studies helped establish the relationships between them and their potential impacts on the results. The main characteristics of each survey were tabulated and presented clearly and objectively. The methodology was systematically addressed with tabulation of data.

The costs and ICERs were adjusted to 2021 US dollars using a web-based tool [29, 30] that uses data from the International Monetary Fund, performing a two-step approach. First, it adjusts the price to a target year, using a Gross Domestic Product deflator index. Second, it converts the adjusted price to a target currency, using Purchasing Power Parities.

## Results

**Fig. 1:**
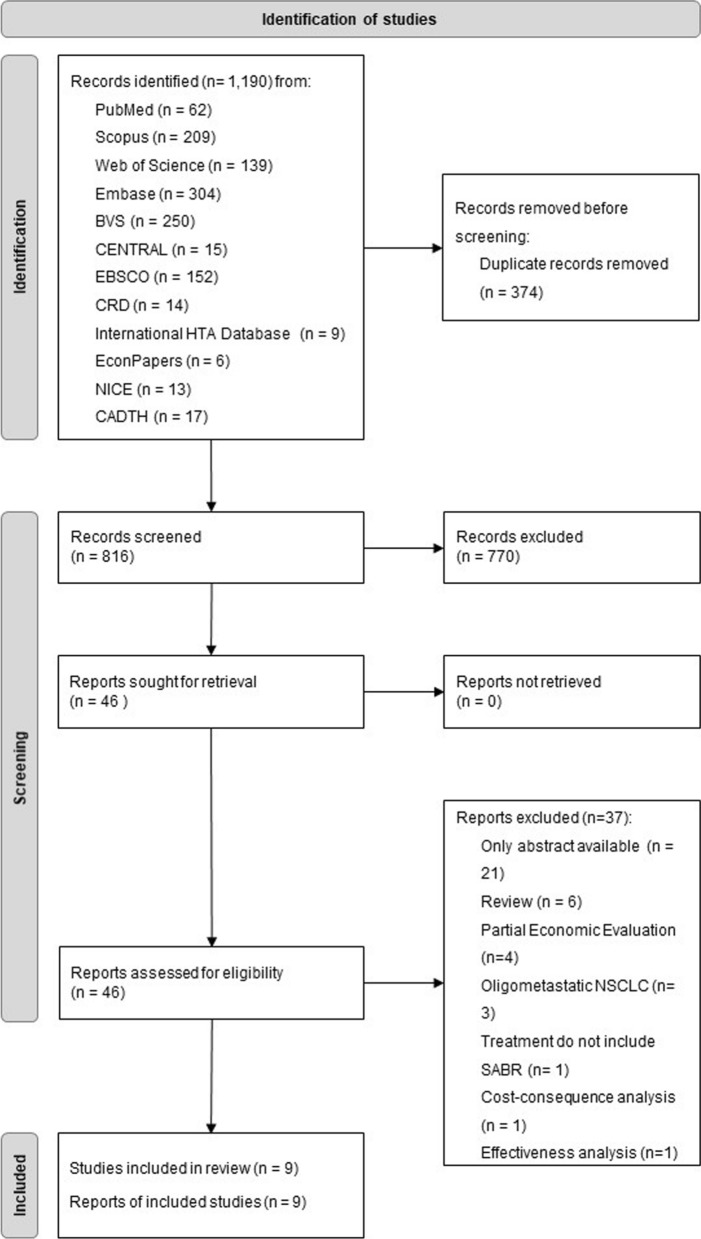
Flow diagram of study identification and selection

The initial search retrieved 1190 records, of which we eradicated 374 duplicates resulting in 816 records that were screened by the title and abstract. 46 reports remained, of which 37 ended excluded, resulting in a total of 9 studies comprised in this analysis (Fig. 1). The included studies are described in Table 2, and the excluded reports and the reasons for exclusion are explained in the supplementary material (Additional file 2).

**Table 2:** Main characteristics of the included studies

Study	Country	Type of study	Study design	Perspective	Type of treatment	Strategies compared	Main results (costs adjusted to 2021 US dollars)	CHEERS properly reported items	Potential conflicts of interest
Grutters et al., 2010 [37]	The Netherlands	CUA	Model-based	Health care payer	Radiotherapy	Inoperable stage I NSCLC: Carbon-ion Proton therapy CRT SABR Operable stage I NSCLC: Carbon-ion Proton therapy SABR	Inoperable stage I NSCLC: SABR dominated proton therapy and CRT Carbon-ion was not cost-effective compared to SABR: ICER $100,081/QALY Operable stage I NSCLC: SABR dominated other techniques	63%	Yes
Sher; Wee; Punglia, 2011 [32]	United States	CUA	Model-based	Health care payer	Radiotherapy	CRT SABR RFA	SABR was cost-effective compared to 3D-CRT: ICER $7,393/ QALY SABR was cost-effective compared to RFA: ICER $17,375/QALY	50%	No
Puri et al., 2012 [31]	United States	CEA	Model-based	Health care payer	Radiotherapy and surgery	Surgical intervention SABR	Surgical intervention was cost-effective compared to SABR: ICER $9,444/LYG	29%	No
Shah et al., 2013 [34]	United States	CUA	Model-based	Health care payer	Radiotherapy and surgery	MOP: SABR Wedge resection COP: SABR Lobectomy	MOP: SABR dominated wedge resection COP: lobectomy was cost-effective compared to SABR: ICER $15,472/QALY	50%	No
Louie et al., 2014 [35]	Canada	CUA	Model-based	Health care payer	Radiotherapy and surgery	CRT SABR Sublobar resection Lobectomy Pneumonectomy BSC	SABR dominated CRT, sublobar resection, and BSC Lobectomy was cost effective compared to SABR: ICER $52,149/ QALY	33%	Yes
Mitera et al., 2014 [35]	Canada	CEA	Single study-based	Health care payer / Hospital	Radiotherapy	CRT SABR	SABR was cost-effective compared to CFRT: ICER $1,110/LYG*. $934/LYG**	42%	Yes
Smith et al., 2015 [33]	United States	CEA	Single study-based	Health care payer	Radiotherapy and surgery	SABR Sublobar resection Lobectomy	Sublobar resection was not cost-effective compared to SABR: ICER $51,585/LYG Lobectomy was cost-effective compared to SABR: ICER $32,345/ LYG	33%	Yes
Paix et al., 2018 [39]	France	CUA	Model-based	Health care payer	Radiotherapy and surgery	SABR Lobectomy	SABR dominated lobectomy	67%	No
Wolff et al., 2020 [39]	The Netherlands	CUA	Model-based	Health care payer	Radiotherapy and surgery	VATS resection SABR	SABR dominated VATS resection	54%	Yes

BSC: Best supportive care, CEA: Cost-effectiveness analysis, CHEERS: Consolidated Health Economic Evaluation Reporting Standards, COP: clearly operable patients, CRT: Conventional radiotherapy, CUA: Cost-utility analysis, ICER: incremental cost-effectiveness ratio, LYG: life years gained, MOP: Marginally operable patients, NSCLC: Non-small cell lung cancer, SABR: Stereotactic ablative radiotherapy, VATS: Video Assisted Thoracic Surgery

^*^Healthcare payer perspective, **Hospital perspective

The studies were made in the United States (n = 4) [31–34], Canada (n = 2) [35, 36], The Netherlands (n = 2) [37, 38], and France (n = 1) [39]. Three of them were cost-effectiveness analyses (CEA) [31, 33, 36], while the other six were cost-utility analyses (CUA) [32, 34, 35, 37–39]. Seven were model-based analyses [31, 32, 34, 35, 37–39], of which five used a decision-analytic Markov model [31, 32, 34, 37, 39]. Two used data from retrospective cohorts [33, 39]. All of them were designed from a healthcare payer perspective; one also addressed the hospital costs perspective [36]. Most of the studies (n = 5) [33, 35–38] have potential conflicts of interest related to manufacturer or funding sources directly from the industry.

Three studies compared exclusively radiotherapy techniques [32, 36, 37], while six compared radiotherapy techniques with surgical procedures [31, 33–35, 38, 39]. In the subgroup that compared only radiotherapy techniques, one of them found SABR to be dominant (more effective and less expensive) compared to other radiotherapy techniques [39]. The other two studies found SABR more costly but more effective, qualified as cost-effective in the analysis made [32, 36]. All of them were in favor of SABR against other radiotherapy techniques.

In the subgroup that compared SABR with surgical procedures, we found conflicting results. The studies that compared SABR to lobectomy found lobectomy to be cost-effective in three [33–35], while SABR dominated in one of them [39]. In comparison with sublobar resection, SABR was dominant in two [34, 35] and cost-effective in another one [33]. Two of the studies analyzed a mix of surgical techniques together [31, 38], and one of them found that surgical intervention was more cost-effective [31]. The other found that SABR dominated surgical procedures [38].

The cost categories in each study are described in Table 3. All of them comprehended only direct healthcare costs. Two did not clearly explain the cost categories encompassed [33, 35]. Radiotherapy delivery and treatment complications were comprised by the seven remaining studies [31, 32, 34, 36–39], while patient transport reimbursement [39] and equipment [36] were included in only one study each.

**Table 3:** Cost components included in the studies

Study	Initial treatment				Pre-treatment and follow-up							General costs	
	Radiotherapy	Surgical procedures	Equipment	Materials and supplies	Ambulatory visits	Medication	Tests	Chemotherapy	Treatment Complications	Palliative Care	Hospitalization	Administrative and overhead	Transport
Grutters et al.,2010 [37]	Included	Not included	Not included	Unclear	Included	Included	Not included	Not included	Included	Not included	Included	Not included	Not included
Sher; Wee; Punglia, 2011 [32]	Included	Not included	Not included	Included	Unclear	Included	Included	Not included	Included	Included	Included	Not included	Not included
Puri et al., 2012 [31]	Included	Included	Unclear	Unclear	Unclear	Unclear	Unclear	Included	Included	Unclear	Unclear	Unclear	Unclear
Shah et al., 2013 [34]	Included	Included	Not included	Included	Unclear	Included	Included	Included	Included	Included	Included	Not included	Not included
Louie et al., 2014 [35]	Unclear	Unclear	Unclear	Unclear	Unclear	Unclear	Unclear	Unclear	Unclear	Unclear	Unclear	Unclear	Unclear
MITERA et al., 2014 [36]	Included	Not included	Included	Included	Not included	Not included	Included	Not included	Included	Not included	Included	Not included	Not included
Smith et al., 2015 [33]	Unclear	Unclear	Unclear	Unclear	Unclear	Unclear	Unclear	Unclear	Unclear	Unclear	Unclear	Unclear	Unclear
Paix et al., 2018 [39]	Included	Included	Not included	Unclear	Included	Included	Included	Included	Included	Included	Unclear	Not included	Included
Wolff et al., 2020 [38]	Included	Included	Not included	Unclear	Included	Unclear	Included	Unclear	Included	Unclear	Included	Not included	Not included

In Table 4, we describe the costs and effectiveness of SABR in each study. The SABR total costs, from the health payer perspective, varied from $7,973 to $63,012. According to health outcomes, the QALY ranged from 1.91 to 16.35, and the LYG ranged from 1.03 to 8.55.

**Table 4:** SABR costs and effectiveness

Study	SABR total cost (2021 US dollars)	SABR effectiveness
Grutters et al., 2010 [37]	Inoperable stage I NSCLC: $20,640 Operable stage I NSCLC: $12,626	Inoperable stage I NSCLC: 2.59 QALY Operable stage I NSCLC: 3.20 QALY
Sher; Wee; Punglia, 2011 [32]	$63,012	1.91 QALY
Puri et al., 2012 [31]	$17,240	2.94 LYG
Shah et al., 2013 [34]	MOP: $49,281 COP: $46,955	MOP: 8.03 QALY COP: 8.21 QALY
Louie et al., 2014 [35]	$8,222	Not described
Mitera et al., 2014 [36]	$7,973* $6,903**	1.03 LYG
Smith et al., 2015 [33]	SABR to sublobar resection: $62,241 SABR to lobectomy: $62,069	SABR to sublobar resection: 3.6 LYG SABR to lobectomy: 3.8 LYG
Paix et al., 2018 [39]	$12,473	16.35 QALY
Wolff et al., 2020 [38]	$27,558	5.86 QALY

COP: clearly operable patients, MOP: Marginally operable patients, LYG: Life years gained, NSCLC: Non-small cell lung cancer, QALY: Quality-adjusted life years, SABR: Stereotactic ablative radiotherapy

^*^Ministry of Health and Long-Term Care perspective

^**^Hospital perspective

**Fig. 2:**
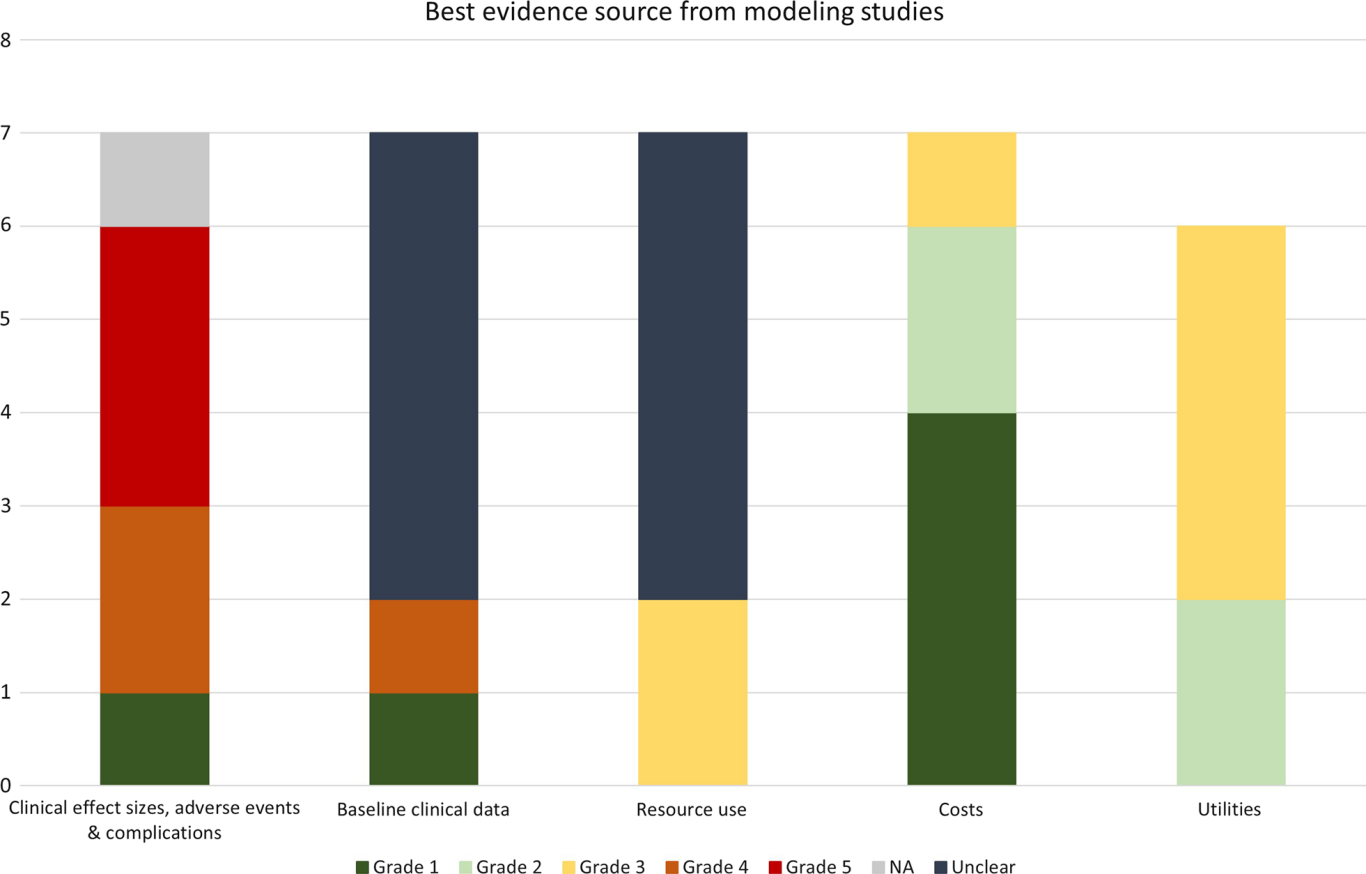
Best evidence sources from modeling studies. One of the modeling studies used life-years gained as an outcome measure; therefore, it did not have a source for utility

The assessment of the quality of evidence used as parameters in the model-based studies is presented in Fig. 2, and its full description can be consulted (Additional file 3). Considering clinical effect size, adverse effects, and complications, only one study [39] was attributed Grade 1 evidence. However, even this work also employed non-analytical studies. The majority mixed data from various sources, including observational studies, non-analytical studies, and experimental single-arm phase II trials. Regarding baseline clinical data, most of them did not elucidate the parameters used. Concerning resource use and costs, the majority did not clarify resource parameters source, although nearly all informed cost unit parameters source. Considering utilities, all of the CUA told the source of this data, most from a previously published study.

Regarding the reporting quality, the proportion of items properly informed varied from 29 to 67%, with an average of 47% and a median of 50% (Table 2, full CHEERS checklist available on Additional file 4). None of the studies unequivocally stated aspects of the health system in which the decision must be made. About clinical effectiveness data, only one thoroughly described the methods used to identify the data sources. Regarding uncertainty, none of them fully reported the effects of uncertainty for all input parameters. All of them stated the broader context and explained the compared strategies and the reasons for their choice.

## Discussion

This systematic review revealed nine full health economic evaluation studies classified into two groups: radiotherapy techniques comparison and SABR vs. surgical techniques comparison.

In the subgroup of studies that compared exclusively radiotherapy techniques, the results suggest SABR as dominant or more cost-effective in treating inoperable early-stage NSCLC. This conclusion aligns with oncology societies' recommendations and healthcare systems protocols [13–16].

In comparison to surgical techniques, the studies did not reach a consensus. Lobectomy seems to be cost-effective or dominant compared to SABR in most of the studies. SABR appears to be cost-effective or dominant compared to less radical surgical techniques. Surgical procedures, such as lobectomy or wedge resection, were more expensive than SABR in all the included studies. The differences across studies results rely on clinical effectiveness data, such as probability of cancer recurrence. As they used different data sources, the results varied according to whether the SABR was more or less effective compared to surgical procedure in the chosen source.

Most of the studies had potential conflicts of interests and, though this does not imply disregarding the results, it should raise an alert on it. Manufacturers' interests in incorporating new technologies could provide biases in study design and report writing.

One major limitation of the studies is that they were all developed from the healthcare payer's perspective rather than the societal perspective. It implies that out-of-pocket costs paid by the patient and indirect costs such as loss of productivity were not considered. Moreover, all of them were conducted in high-income countries, so there are implications about the transferability of the results to upper-middle and lower-middle-income countries.

First, developed and developing countries have different epidemiological realities, with higher lung cancer incidence and mortality in developed countries. Nevertheless, this aspect may change with higher smokers’ concentrations in developing countries [1].

Second, there is a substantial difference between the healthcare system organization and funding. Although upper-middle and lower-middle-income countries have high growth rates of per capita health spending, the absolute values are quite different. In 2016, high-income countries spent $5,252 while upper-middle and lower-middle expended $491 and $81, respectively [40]. That said, the willingness to pay threshold should also be considerably different in these countries.

Third, the costs of SABR adoption may be considerably higher in developing countries than reported by these studies, as they did not address some issues about the implementation of the technique. All of them considered that the SABR technique was already spread. Only one of the studies included acquisition and maintenance costs, but merely to obtain more accurate procedure pricing. Therefore, the SABR might be less cost-effective in developing countries than reported by these studies.

In a resource-limited scenario, it is necessary to consider some issues about the program's plan, the initiation of the adoption, and its sustainability over time [41]. The new technique may demand equipment or accessories that are not currently available at healthcare facilities. The acquisition and installation of these items must be regarded. Occasionally, it also involves changes in physical structure, as facilities renovation or an increase in power supply, which should also be considered.

Personal training to use the equipment is also required to deliver the new technique properly. Both the healthcare professionals and the maintenance teams should undergo training courses, eventually repeated over time due to professional turnover.

Finally, the new technique adoption must also consider the location of the equipment. It is fundamental to adequate its capacity to the estimated number of procedures performed and patients treated yearly. It is also advisable to evaluate whether the equipment can be used to treat other diseases. The capacity data should be correlated with epidemiological indexes to advise its adoption and placement properly.

Another issue we found was a substantial difference in the total costs of SABR. It is common knowledge that the US health system has higher healthcare delivery costs than other high-income countries [42]. The three most expensive results are from the US studies [32–34], and the costs varied more than 20% among them. As the methodologies to cost calculations were not clearly described, we could not assess the reasons for such disparity.

Two of the included studies did not clarify what costs participated in their analyses [33, 35]. Apart from that, the remaining seven studies were highly heterogenous on the cost categories considered. This heterogeneity compromises the comparison of them, as there was no pattern on cost inclusion criteria. Crucial costs, such as the capital cost of the linear accelerator, which is essential to radiotherapy delivery, or the costs related to outpatient follow-up, were not considered in a considerable part of the studies. The absence of a minimum standard of included costs makes it impossible to understand the real cause of the variation across studies.

Considering the quality of the evidence used, most of the model-based studies used evidence rated as 4 or 5 at crucial parameters, such as survival rates or local control rate [32, 34, 35, 37, 38]. Only one of the studies used randomized clinical trial data [39], and even this source has some issues, as it is a pooled analysis of two different studies closed due to slow accrual. Most of them also included experimental single-arm phase II data [32, 34, 35, 37, 39]. Thus, one of the most critical parameters is not trustworthy.

Although most of the studies justify the above due to scarcity of high-quality evidence, this issue cannot be entirely managed in sensitivity analysis and could impact the results of the studies. Using systematic review to obtain a more accurate overview of the clinical effectiveness data would be a better approach to this issue. Nevertheless, only one of the studies [37] did it.

Most of the studies did not fully explain the resources used and how they attained total costs for the compared techniques. Some of them did not list the items considered in the analysis. The ones that did so failed to explain how they got these parameters. Although nearly all of them correctly identified the source for cost units (e.g., reimbursement tables), it was impossible to evaluate if the costs included were appropriate for the analyses.

The studies that measured QALY mostly used data from previous studies. However, these sources were not specifically about early-stage but from an advanced NSCLC perspective, including chemotherapy and its adverse effects. The cancer stage and treatment delivered might have substantially different effects on quality-of-life measures.

Finally, one last major issue in these studies was poor reporting quality. Most of the reports had at least half of the items of the CHEERS checklist not correctly described. One key element of transferability analysis, the description of the system in which the decision needs to be made, was not related in any of them. The missing information jeopardized the transferability analysis, as we cannot critically appraise substantive elements of the studies.

This work managed to assess the published full EEs on SABR for the treatment of early-stage NSCLC. These results, however, did not include studies that were only published as a poster presentation at congresses and conferences, and it is a limitation of this work. Nevertheless, it is unlikely that the unpublished work would have better methodological strictness than the published ones.

Further works on this area should address some issues that we found inappropriate on the reviewed studies. First, the researchers should search for robust evidence to use as model parameters. If it is not possible to find high-quality evidence, it should be clearly stated, and the chosen parameters be justified. Second, regarding resources used and costs, the studies should describe all the cost components included and how the resources were identified and quantified. Third, we believe that radiotherapy economic evaluation studies should include at least the following cost categories: radiotherapy delivery costs, capital costs of equipment, materials and supplies, ambulatory visits, medications, tests, treatment complications, palliative care, and hospitalization. Last, but not least, the published reports should adhere to CHEERS guidelines, as it addresses essential aspects to comprehend the studies developed.

## Conclusions

The included studies suggest that SABR is dominant or more cost-effective and should be the first-choice radiotherapy technique to early-stage NSCLC but do not reach a unanimous decision about its use compared to surgical procedures. The included studies lacked methodological information, making it impossible to assess transferability to other scenarios. Some economic aspects related to the technique adoption were not addressed, risking underestimating SABR's total costs. Thus, we strongly recommend that upper-middle and lower-middle-income countries develop CUA and CEA in their own context.

## Supplementary Information

**Additional file 1. **Full Search Strategy.

**Additional file 2. **Excluded reports.

**Additional file 3. **Quality appraisal of the evidence of the study.

**Additional file 4. **CHEERS checklist assessment.

## Data Availability

All data relevant to the study are included in the article or uploaded as supplementary information.

## Abbreviations

BRISA: Regional Health Technology Assessment Reports of the Americas
CADHT: Canadian Agency for Drugs and Technologies in Health
CEA: Cost-effectiveness analyses
CHEERS: Consolidated Health Economic Evaluation Reporting Standards Statement
CI: Confidence interval
CONITEC: National Commission for the Incorporation of Technologies in SUS
CRT: Conventional radiotherapy
CUA: Cost-utility analyses
EE: Economic evaluations
FEV1: Forced expiratory volume in the first second
HR: Hazard ratio
NICE: The National Institute for Health and Care Excellence
NSCLC: Non-small cell lung cancer
PRISMA: Preferred reporting items for systematic reviews and meta-analyzes
SABR: Stereotactic ablative radiotherapy
SBRT: Stereotactic body radiation therapy
