# Supplementary material for: Systematic review of economic evaluations on stereotactic ablative radiotherapy (SABR) compared to other radiotherapy techniques or surgical procedures for early-stage non-small cell lung cancer
Source: Cost Eff Resour Alloc. 2023 Jan 16;21:4. doi: 10.1186/s12962-023-00415-1 (PMC9841623; doi:10.1186/s12962-023-00415-1)
Supplement: Supplementary file 1 — Additional file 1. Full Search Strategy. [file 12962_2023_415_MOESM1_ESM.docx]

**Additional file 1– Full Search Strategy**

| **Database** | **Search terms** | **Records Identified** | |
| --- | --- | --- | --- |
| **MedLine via PubMed** | (("Radiosurgery"[Title/Abstract] OR "CyberKnife Radiosurgeries"[Title/Abstract] OR "CyberKnife Radiosurgery"[Title/Abstract] OR "Gamma Knife Radiosurgeries"[Title/Abstract] OR "Gamma Knife Radiosurgery"[Title/Abstract] OR "LINAC Radiosurgeries"[Title/Abstract] OR "LINAC Radiosurgery"[Title/Abstract] OR "Linear Accelerator Radiosurgeries"[Title/Abstract] OR "Linear Accelerator Radiosurgery"[Title/Abstract] OR "Radiation Therapy, Stereotactic"[Title/Abstract] OR "Radiation, Stereotactic"[Title/Abstract] OR "Radiosurgery, CyberKnife"[Title/Abstract] OR "Radiosurgery, Gamma Knife"[Title/Abstract] OR "Radiosurgery, LINAC"[Title/Abstract] OR "Radiosurgery, Linear Accelerator"[Title/Abstract] OR "Radiosurgery, Stereotactic"[Title/Abstract] OR "Radiotherapy, Stereotactic Body"[Title/Abstract] OR "Stereotactic Body Radiotherapies"[Title/Abstract] OR "Stereotactic Body Radiotherapy"[Title/Abstract] OR "Stereotactic Radiation"[Title/Abstract] OR "Stereotactic Radiation Therapies"[Title/Abstract] OR "Stereotactic Radiation Therapy"[Title/Abstract] OR "Stereotactic Radiations"[Title/Abstract] OR "Stereotactic Radiosurgeries"[Title/Abstract] OR "Stereotactic Radiosurgery"[Title/Abstract] OR "Therapy, Stereotactic Radiation"[Title/Abstract] OR "radiofrequency ablation"[Title/Abstract] OR "stereotactic body radiation therapy"[Title/Abstract] OR "radio-surgery"[Title/Abstract] OR "SABR"[Title/Abstract] OR "SABRT"[Title/Abstract] OR "SBRT"[Title/Abstract] OR "stereotactic ablative body radiation therapy"[Title/Abstract] OR "stereotactic ablative body radiotherapy"[Title/Abstract] OR "stereotactic ablative radiation therapy"[Title/Abstract] OR "stereotactic ablative radiotherapy"[Title/Abstract] OR "stereotaxic ablative radiation therapy"[Title/Abstract] OR "stereotaxic ablative radiotherapy"[Title/Abstract] OR "stereotaxic body radiation therapy"[Title/Abstract] OR "stereotaxic body radiotherapy"[Title/Abstract] OR "SRS (stereotactic radiosurgery)"[Title/Abstract] OR "stereotactic radio-surgery"[Title/Abstract] OR "stereotaxic radio-surgery"[Title/Abstract] OR "stereotaxic radiosurgery"[Title/Abstract]) AND (("cost*"[Title/Abstract] OR "costs and cost analysis"[MeSH:noexp] OR "cost benefit analys*"[Title/Abstract] OR "cost benefit analysis"[MeSH] OR "health care costs"[MeSH:noexp]) OR ("Cost-Benefit Analysis"[Title/Abstract] OR "Benefits[Title/Abstract] AND Costs"[Title/Abstract] OR "Cost Benefit"[Title/Abstract] OR "Cost Benefit Analysis"[Title/Abstract] OR "Cost Effectiveness"[Title/Abstract] OR "Cost-Benefit Data"[Title/Abstract] OR "Cost-Effectiveness Analysis"[Title/Abstract] OR "Cost-Utility Analysis"[Title/Abstract] OR "Costs[Title/Abstract] AND Benefits"[Title/Abstract] OR "Economic Evaluation"[Title/Abstract] OR "Marginal Analysis"[Title/Abstract] OR "Cost Efficiency Analysis"[Title/Abstract] OR "Cost-Effectiveness Evaluation"[Title/Abstract] OR "Costs[Title/Abstract] AND Cost Analysis"[Title/Abstract] OR "Affordability"[Title/Abstract] OR "Analysis, Cost"[Title/Abstract] OR "Cost"[Title/Abstract] OR "Cost Analysis"[Title/Abstract] OR "Cost Comparison"[Title/Abstract] OR "Cost Measures"[Title/Abstract] OR "Cost-Minimization Analysis"[Title/Abstract] OR "Costs[Title/Abstract] AND Cost Analyses"[Title/Abstract] OR "Costs, Cost Analysis"[Title/Abstract] OR "Pricing"[Title/Abstract] OR "Health Economics"[Title/Abstract] OR "Health Evaluation"[Title/Abstract] OR "Economic Assessment in Health"[Title/Abstract] OR "Economic Assessment in Health Care"[Title/Abstract] OR "Economic Assessment in Healthcare"[Title/Abstract] OR "Economic Evaluation in Health"[Title/Abstract] OR "Economic Evaluation in Health Care"[Title/Abstract] OR "Economic Evaluation in Healthcare"[Title/Abstract] OR "Analyses, Cost-Benefit"[Title/Abstract] OR "Analysis, Cost-Benefit"[Title/Abstract] OR "Cost-Benefit Analyses"[Title/Abstract] OR "Analyses, Cost Benefit"[Title/Abstract] OR "Analysis, Cost Benefit"[Title/Abstract] OR "Cost Benefit Analyses"[Title/Abstract] OR "Effectiveness, Cost"[Title/Abstract] OR "Cost Benefit Data"[Title/Abstract] OR "Data, Cost-Benefit"[Title/Abstract] OR "Analyses, Cost-Utility"[Title/Abstract] OR "Analysis, Cost-Utility"[Title/Abstract] OR "Cost Utility Analysis"[Title/Abstract] OR "Cost-Utility Analyses"[Title/Abstract] OR "Economic Evaluation"[Title/Abstract] OR "Economic Evaluations"[Title/Abstract] OR "Evaluation, Economic"[Title/Abstract] OR "Evaluations, Economic"[Title/Abstract] OR "Analyses, Marginal"[Title/Abstract] OR "Analysis, Marginal"[Title/Abstract] OR "Marginal Analyses"[Title/Abstract] OR "Analysis, Cost-Effectiveness"[Title/Abstract] OR "Cost Effectiveness Analysis"[Title/Abstract] OR "Cost, Cost Analysis"[Title/Abstract] OR "Analyses, Cost"[Title/Abstract] OR "Cost Analyses"[Title/Abstract] OR "Comparison, Cost"[Title/Abstract] OR "Comparisons, Cost"[Title/Abstract] OR "Cost Comparisons"[Title/Abstract] OR "Affordabilities"[Title/Abstract] OR "Analyses, Cost-Minimization"[Title/Abstract] OR "Analysis, Cost-Minimization"[Title/Abstract] OR "Cost Minimization Analysis"[Title/Abstract] OR "Cost-Minimization Analyses"[Title/Abstract] OR "Costs"[Title/Abstract] OR "Cost Measure"[Title/Abstract] OR "Measure, Cost"[Title/Abstract] OR "Measures, Cost"[Title/Abstract] OR "Health Care Costs"[Title/Abstract] OR "Cost, Health Care"[Title/Abstract] OR "Costs, Health Care"[Title/Abstract] OR "Health Care Cost"[Title/Abstract] OR "Health Costs"[Title/Abstract] OR "Cost, Health"[Title/Abstract] OR "Costs, Health"[Title/Abstract] OR "Health Cost"[Title/Abstract] OR "Healthcare Costs"[Title/Abstract] OR "Cost, Healthcare"[Title/Abstract] OR "Costs, Healthcare"[Title/Abstract] OR "Healthcare Cost"[Title/Abstract] OR "Medical Care Costs"[Title/Abstract] OR "Costs, Medical Care"[Title/Abstract] OR "Cost, Medical Care"[Title/Abstract] OR "Medical Care Cost"[Title/Abstract] OR "Treatment Costs"[Title/Abstract] OR "Cost, Treatment"[Title/Abstract] OR "Costs, Treatment"[Title/Abstract] OR "Treatment Cost"[Title/Abstract] OR "cost control"[Title/Abstract] OR "cost of illness"[Title/Abstract] OR " cost effectiveness ratio"[Title/Abstract] OR "cost benefit ratio"[Title/Abstract] OR "audit, cost"[Title/Abstract] OR "cost audit"[Title/Abstract] OR "cost containment"[Title/Abstract] OR "cost savings"[Title/Abstract] OR "cost minimization"[Title/Abstract] OR "cost of illness analysis"[Title/Abstract] OR "economic aspects of illness"[Title/Abstract] OR "cost utility"[Title/Abstract]))) AND ("Lung Neoplasms"[Title/Abstract] OR "Cancer of Lung"[Title/Abstract] OR "Cancer of the Lung"[Title/Abstract] OR "cancer lung"[Title/Abstract] OR "cancer pulmonary"[Title/Abstract] OR "cancers lung"[Title/Abstract] OR "cancers pulmonary"[Title/Abstract] OR "Lung Cancer"[Title/Abstract] OR "Lung Cancers"[Title/Abstract] OR "Lung Neoplasm"[Title/Abstract] OR "neoplasm lung"[Title/Abstract] OR "neoplasm pulmonary"[Title/Abstract] OR "neoplasms lung"[Title/Abstract] OR "neoplasms pulmonary"[Title/Abstract] OR "Pulmonary Cancer"[Title/Abstract] OR "Pulmonary Cancers"[Title/Abstract] OR "Pulmonary Neoplasm"[Title/Abstract] OR "Pulmonary Neoplasms"[Title/Abstract] OR "Adenocarcinoma of Lung"[Title/Abstract] OR "Lung Adenocarcinoma"[Title/Abstract] OR "carcinoma non small cell lung"[Title/Abstract] OR "carcinoma non small cell lung"[Title/Abstract] OR "Non-Small Cell Lung Cancer"[Title/Abstract] OR "Non-Small-Cell Lung Carcinoma"[Title/Abstract] OR "Nonsmall Cell Lung Cancer"[Title/Abstract]) | 62 | |
| **EMBASE** | ('lung neoplasms':ab,ti OR 'cancer of lung':ab,ti OR 'cancer of the lung':ab,ti OR 'cancer lung':ab,ti OR 'cancer pulmonary':ab,ti OR 'cancers lung':ab,ti OR 'cancers pulmonary':ab,ti OR 'lung cancer':ab,ti OR 'lung cancers':ab,ti OR 'lung neoplasm':ab,ti OR 'neoplasm lung':ab,ti OR 'neoplasm pulmonary':ab,ti OR 'neoplasms lung':ab,ti OR 'neoplasms pulmonary':ab,ti OR 'pulmonary cancer':ab,ti OR 'pulmonary cancers':ab,ti OR 'pulmonary neoplasm':ab,ti OR 'pulmonary neoplasms':ab,ti OR 'adenocarcinoma of lung':ab,ti OR 'lung adenocarcinoma':ab,ti OR 'carcinoma non small cell lung':ab,ti OR 'non-small cell lung cancer':ab,ti OR 'non-small-cell lung carcinoma':ab,ti OR 'nonsmall cell lung cancer':ab,ti) AND ('radiosurgery':ab,ti OR 'cyberknife radiosurgeries':ab,ti OR 'cyberknife radiosurgery':ab,ti OR 'gamma knife radiosurgeries':ab,ti OR 'gamma knife radiosurgery':ab,ti OR 'linac radiosurgeries':ab,ti OR 'linac radiosurgery':ab,ti OR 'linear accelerator radiosurgeries':ab,ti OR 'linear accelerator radiosurgery':ab,ti OR 'radiation therapy, stereotactic':ab,ti OR 'radiation, stereotactic':ab,ti OR 'radiosurgery, cyberknife':ab,ti OR 'radiosurgery, gamma knife':ab,ti OR 'radiosurgery, linac':ab,ti OR 'radiosurgery, linear accelerator':ab,ti OR 'radiosurgery, stereotactic':ab,ti OR 'radiotherapy, stereotactic body':ab,ti OR 'stereotactic body radiotherapies':ab,ti OR 'stereotactic body radiotherapy':ab,ti OR 'stereotactic radiation':ab,ti OR 'stereotactic radiation therapies':ab,ti OR 'stereotactic radiation therapy':ab,ti OR 'stereotactic radiations':ab,ti OR 'stereotactic radiosurgeries':ab,ti OR 'stereotactic radiosurgery':ab,ti OR 'therapy, stereotactic radiation':ab,ti OR 'radiofrequency ablation':ab,ti OR 'stereotactic body radiation therapy':ab,ti OR 'radio-surgery':ab,ti OR 'sabr':ab,ti OR 'sabrt':ab,ti OR 'sbrt':ab,ti OR 'stereotactic ablative body radiation therapy':ab,ti OR 'stereotactic ablative body radiotherapy':ab,ti OR 'stereotactic ablative radiation therapy':ab,ti OR 'stereotactic ablative radiotherapy':ab,ti OR 'stereotaxic ablative radiation therapy':ab,ti OR 'stereotaxic ablative radiotherapy':ab,ti OR 'stereotaxic body radiation therapy':ab,ti OR 'stereotaxic body radiotherapy':ab,ti OR 'srs stereotactic radiosurgery':ab,ti OR 'stereotactic radio-surgery':ab,ti OR 'stereotaxic radio-surgery':ab,ti OR 'stereotaxic radiosurgery':ab,ti) AND ('cost-benefit analysis':ab,ti OR 'benefits':ab,ti OR 'cost benefit':ab,ti OR 'cost benefit analysis':ab,ti OR 'cost effectiveness':ab,ti OR 'cost-benefit data':ab,ti OR 'cost-effectiveness analysis':ab,ti OR 'cost-utility analysis':ab,ti OR 'marginal analysis':ab,ti OR 'cost efficiency analysis':ab,ti OR 'cost-effectiveness evaluation':ab,ti OR 'affordability':ab,ti OR 'analysis, cost':ab,ti OR 'cost':ab,ti OR 'cost analysis':ab,ti OR 'cost comparison':ab,ti OR 'cost measures':ab,ti OR 'cost-minimization analysis':ab,ti OR 'costs, cost analysis':ab,ti OR 'pricing':ab,ti OR 'health economics':ab,ti OR 'health evaluation':ab,ti OR 'economic assessment in health':ab,ti OR 'economic assessment in health care':ab,ti OR 'economic assessment in healthcare':ab,ti OR 'economic evaluation in health':ab,ti OR 'economic evaluation in health care':ab,ti OR 'economic evaluation in healthcare':ab,ti OR 'analyses, cost-benefit':ab,ti OR 'analysis, cost-benefit':ab,ti OR 'cost-benefit analyses':ab,ti OR 'analyses, cost benefit':ab,ti OR 'analysis, cost benefit':ab,ti OR 'cost benefit analyses':ab,ti OR 'effectiveness, cost':ab,ti OR 'cost benefit data':ab,ti OR 'data, cost-benefit':ab,ti OR 'analyses, cost-utility':ab,ti OR 'analysis, cost-utility':ab,ti OR 'cost utility analysis':ab,ti OR 'cost-utility analyses':ab,ti OR 'economic evaluation':ab,ti OR 'economic evaluations':ab,ti OR 'evaluation, economic':ab,ti OR 'evaluations, economic':ab,ti OR 'analyses, marginal':ab,ti OR 'analysis, marginal':ab,ti OR 'marginal analyses':ab,ti OR 'analysis, cost-effectiveness':ab,ti OR 'cost effectiveness analysis':ab,ti OR 'cost, cost analysis':ab,ti OR 'analyses, cost':ab,ti OR 'cost analyses':ab,ti OR 'comparison, cost':ab,ti OR 'comparisons, cost':ab,ti OR 'cost comparisons':ab,ti OR 'affordabilities':ab,ti OR 'analyses, cost-minimization':ab,ti OR 'analysis, cost-minimization':ab,ti OR 'cost minimization analysis':ab,ti OR 'cost-minimization analyses':ab,ti OR 'costs':ab,ti OR 'cost measure':ab,ti OR 'measure, cost':ab,ti OR 'measures, cost':ab,ti OR 'health care costs':ab,ti OR 'cost, health care':ab,ti OR 'costs, health care':ab,ti OR 'health care cost':ab,ti OR 'health costs':ab,ti OR 'cost, health':ab,ti OR 'costs, health':ab,ti OR 'health cost':ab,ti OR 'healthcare costs':ab,ti OR 'cost, healthcare':ab,ti OR 'costs, healthcare':ab,ti OR 'healthcare cost':ab,ti OR 'medical care costs':ab,ti OR 'costs, medical care':ab,ti OR 'cost, medical care':ab,ti OR 'medical care cost':ab,ti OR 'treatment costs':ab,ti OR 'cost, treatment':ab,ti OR 'costs, treatment':ab,ti OR 'treatment cost':ab,ti OR 'cost control':ab,ti OR 'cost of illness':ab,ti OR 'cost effectiveness ratio':ab,ti OR 'cost benefit ratio':ab,ti OR 'audit, cost':ab,ti OR 'cost audit':ab,ti OR 'cost containment':ab,ti OR 'cost savings':ab,ti OR 'cost minimization':ab,ti OR 'cost of illness analysis':ab,ti OR 'economic aspects of illness':ab,ti OR 'cost utility':ab,ti) | 304 | |
| **Cochrane Library / Central** | #1: (“Radiosurgery” OR “CyberKnife Radiosurgeries” OR “CyberKnife Radiosurgery” OR “Gamma Knife Radiosurgeries” OR “Gamma Knife Radiosurgery” OR “LINAC Radiosurgeries” OR “LINAC Radiosurgery” OR “Linear Accelerator Radiosurgeries” OR “Linear Accelerator Radiosurgery” OR “Radiation Therapy, Stereotactic” OR “Radiation, Stereotactic” OR “Radiosurgery, CyberKnife” OR “Radiosurgery, Gamma Knife” OR “Radiosurgery, LINAC” OR “Radiosurgery, Linear Accelerator” OR “Radiosurgery, Stereotactic” OR “Radiotherapy, Stereotactic Body” OR “Stereotactic Body Radiotherapies” OR “Stereotactic Body Radiotherapy” OR “Stereotactic Radiation” OR “Stereotactic Radiation Therapies” OR “Stereotactic Radiation Therapy” OR “Stereotactic Radiations” OR “Stereotactic Radiosurgeries” OR “Stereotactic Radiosurgery” OR “Therapy, Stereotactic Radiation” OR “radiofrequency ablation” OR “stereotactic body radiation therapy” OR “radio-surgery” OR “SABR” OR “SABRT” OR “SBRT” OR “stereotactic ablative body radiation therapy” OR “stereotactic ablative body radiotherapy” OR “stereotactic ablative radiation therapy” OR “stereotactic ablative radiotherapy” OR “stereotaxic ablative radiation therapy” OR “stereotaxic ablative radiotherapy” OR “stereotaxic body radiation therapy” OR “stereotaxic body radiotherapy” OR “SRS (stereotactic radiosurgery)” OR “stereotactic radio-surgery” OR “stereotaxic radio-surgery” OR “stereotaxic radiosurgery”):ti,ab,kw (Word variations have been searched)  #2: "Lung Neoplasms" OR "Cancer of Lung" OR "Cancer of the Lung" OR "cancer lung" OR "cancer pulmonary" OR "cancers lung" OR "cancers pulmonary" OR "Lung Cancer" OR "Lung Cancers" OR "Lung Neoplasm" OR "neoplasm lung" OR "neoplasm pulmonary" OR "neoplasms lung" OR "neoplasms pulmonary" OR "Pulmonary Cancer" OR "Pulmonary Cancers" OR "Pulmonary Neoplasm" OR "Pulmonary Neoplasms" OR "Adenocarcinoma of Lung" OR "Lung Adenocarcinoma" OR "carcinoma non small cell lung" OR "carcinoma non small cell lung" OR "Non-Small Cell Lung Cancer" OR "Non-Small-Cell Lung Carcinoma" OR "Nonsmall Cell Lung Cancer"  #3: "Costs" OR "Cost Analysis" OR "Affordability" OR "analyses cost" OR "analyses cost minimization" OR "analysis cost" OR "analysis cost minimization" OR "comparison cost" OR "comparisons cost" OR "Cost" OR "Cost Analyses" OR "Cost Analysis" OR "Cost Comparison" OR "Cost Comparisons" OR "Cost Measure" OR "Cost Measures" OR "cost minimization analysis" OR "cost cost analysis" OR "Cost-Minimization Analyses" OR "cost minimization analysis" OR "Costs" OR "costs cost analysis" OR "measure cost" OR "measures cost" OR "Pricing" OR "cost benefit analysis" OR "analyses cost benefit" OR "analyses cost benefit" OR "analyses cost utility" OR "analyses marginal" OR "analysis cost benefit" OR "analysis cost benefit" OR "analysis cost effectiveness" OR "analysis cost utility" OR "analysis marginal" OR "Cost Benefit" OR "cost benefit analyses" OR "cost benefit analysis" OR "cost benefit data" OR "Cost Effectiveness" OR "cost effectiveness analysis" OR "cost utility analysis" OR "cost benefit analyses" OR "cost benefit data" OR "cost effectiveness analysis" OR "Cost-Utility Analyses" OR "cost utility analysis" OR "data cost benefit" OR "Economic Evaluation" OR "Economic Evaluations" OR "effectiveness cost" OR "evaluation economic" OR "evaluations economic" OR "Marginal Analyses" OR "Marginal Analysis"  #4: #1 AND #2 AND #3 | 15 | |
| **BVS (included BRISA)** | ((“lung neoplasms” OR “cancer of lung” OR “cancer of the lung” OR “cancer lung” OR “cancer pulmonary” OR “cancers lung” OR “cancers pulmonary” OR “lung cancer” OR “lung cancers” OR “lung neoplasm” OR “neoplasm lung” OR “neoplasm pulmonary” OR “neoplasms lung” OR “neoplasms pulmonary” OR “pulmonary cancer” OR “pulmonary cancers” OR “pulmonary neoplasm” OR “pulmonary neoplasms” OR “adenocarcinoma of lung” OR “lung adenocarcinoma” OR “carcinoma non small cell lung” OR “non-small cell lung cancer” OR “non-small-cell lung carcinoma” OR “nonsmall cell lung cancer”) ) AND ((“radiosurgery” OR “cyberknife radiosurgeries” OR “cyberknife radiosurgery” OR “gamma knife radiosurgeries” OR “gamma knife radiosurgery” OR “linac radiosurgeries” OR “linac radiosurgery” OR “linear accelerator radiosurgeries” OR “linear accelerator radiosurgery” OR “radiation therapy, stereotactic” OR “radiation, stereotactic” OR “radiosurgery, cyberknife” OR “radiosurgery, gamma knife” OR “radiosurgery, linac” OR “radiosurgery, linear accelerator” OR “radiosurgery, stereotactic” OR “radiotherapy, stereotactic body” OR “stereotactic body radiotherapies” OR “stereotactic body radiotherapy” OR “stereotactic radiation” OR “stereotactic radiation therapies” OR “stereotactic radiation therapy” OR “stereotactic radiations” OR “stereotactic radiosurgeries” OR “stereotactic radiosurgery” OR “therapy, stereotactic radiation” OR “radiofrequency ablation” OR “stereotactic body radiation therapy” OR “radio-surgery” OR “sabr” OR “sabrt” OR “sbrt” OR “stereotactic ablative body radiation therapy” OR “stereotactic ablative body radiotherapy” OR “stereotactic ablative radiation therapy” OR “stereotactic ablative radiotherapy” OR “stereotaxic ablative radiation therapy” OR “stereotaxic ablative radiotherapy” OR “stereotaxic body radiation therapy” OR “stereotaxic body radiotherapy” OR “srs stereotactic radiosurgery” OR “stereotactic radio-surgery” OR “stereotaxic radio-surgery” OR “stereotaxic radiosurgery”) ) AND ("Costs" OR "Cost Analysis" OR "Affordability" OR "analyses cost" OR "analyses cost minimization" OR "analysis cost" OR "analysis cost minimization" OR "comparison cost" OR "comparisons cost" OR "Cost" OR "Cost Analyses" OR "Cost Analysis" OR "Cost Comparison" OR "Cost Comparisons" OR "Cost Measure" OR "Cost Measures" OR "cost minimization analysis" OR "cost cost analysis" OR "Cost-Minimization Analyses" OR "cost minimization analysis" OR "Costs" OR "costs cost analysis" OR "measure cost" OR "measures cost" OR "Pricing" OR "cost benefit analysis" OR "analyses cost benefit" OR "analyses cost benefit" OR "analyses cost utility" OR "analyses marginal" OR "analysis cost benefit" OR "analysis cost benefit" OR "analysis cost effectiveness" OR "analysis cost utility" OR "analysis marginal" OR "Cost Benefit" OR "cost benefit analyses" OR "cost benefit analysis" OR "cost benefit data" OR "Cost Effectiveness" OR "cost effectiveness analysis" OR "cost utility analysis" OR "cost benefit analyses" OR "cost benefit data") | 250 | |
| **LILACS** | ("Câncer Pulmonar" OR "Câncer de Pulmão" OR "Câncer do Pulmão" OR "Neoplasia Pulmonar") AND ("Radioterapia estereotáxica" OR "Radioterapia Estereotática" OR "Radiocirurgia" OR "SABR" OR "SBRT") AND ("Custos" OR "Custo-efetividade" OR "avaliação econômica" OR "Custo" OR "avaliação de tecnologia" ) | 0 | |
| **EBSCO (included CINAHL, Academic Search Premier, Regional Business News)** | ("Lung Neoplasms" OR "Cancer of Lung" OR "Cancer of the Lung" OR "cancer lung" OR "cancer pulmonary" OR "cancers lung" OR "cancers pulmonary" OR "Lung Cancer" OR "Lung Cancers" OR "Lung Neoplasm" OR "neoplasm lung" OR "neoplasm pulmonary" OR "neoplasms lung" OR "neoplasms pulmonary" OR "Pulmonary Cancer" OR "Pulmonary Cancers" OR "Pulmonary Neoplasm" OR "Pulmonary Neoplasms" OR "Adenocarcinoma of Lung" OR "Lung Adenocarcinoma" OR "carcinoma non small cell lung" OR "carcinoma non small cell lung" OR "Non-Small Cell Lung Cancer" OR "Non-Small-Cell Lung Carcinoma" OR "Nonsmall Cell Lung Cancer") AND (“Radiosurgery” OR “CyberKnife Radiosurgeries” OR “CyberKnife Radiosurgery” OR “Gamma Knife Radiosurgeries” OR “Gamma Knife Radiosurgery” OR “LINAC Radiosurgeries” OR “LINAC Radiosurgery” OR “Linear Accelerator Radiosurgeries” OR “Linear Accelerator Radiosurgery” OR “Radiation Therapy, Stereotactic” OR “Radiation, Stereotactic” OR “Radiosurgery, CyberKnife” OR “Radiosurgery, Gamma Knife” OR “Radiosurgery, LINAC” OR “Radiosurgery, Linear Accelerator” OR “Radiosurgery, Stereotactic” OR “Radiotherapy, Stereotactic Body” OR “Stereotactic Body Radiotherapies” OR “Stereotactic Body Radiotherapy” OR “Stereotactic Radiation” OR “Stereotactic Radiation Therapies” OR “Stereotactic Radiation Therapy” OR “Stereotactic Radiations” OR “Stereotactic Radiosurgeries” OR “Stereotactic Radiosurgery” OR “Therapy, Stereotactic Radiation” OR “radiofrequency ablation” OR “stereotactic body radiation therapy” OR “radio-surgery” OR “SABR” OR “SABRT” OR “SBRT” OR “stereotactic ablative body radiation therapy” OR “stereotactic ablative body radiotherapy” OR “stereotactic ablative radiation therapy” OR “stereotactic ablative radiotherapy” OR “stereotaxic ablative radiation therapy” OR “stereotaxic ablative radiotherapy” OR “stereotaxic body radiation therapy” OR “stereotaxic body radiotherapy” OR “SRS (stereotactic radiosurgery)” OR “stereotactic radio-surgery” OR “stereotaxic radio-surgery” OR “stereotaxic radiosurgery”) AND (“Cost-Benefit Analysis” OR “Benefits and Costs” OR “Cost Benefit” OR “Cost Benefit Analysis” OR “Cost Effectiveness” OR “Cost-Benefit Data” OR “Cost-Effectiveness Analysis” OR “Cost-Utility Analysis” OR “Costs and Benefits” OR “Economic Evaluation” OR “Marginal Analysis” OR “Cost Efficiency Analysis” OR “Cost-Effectiveness Evaluation” OR “Costs and Cost Analysis” OR “Affordability” OR “Analysis, Cost” OR “Cost” OR “Cost Analysis” OR “Cost Comparison” OR “Cost Measures” OR “Cost-Minimization Analysis” OR “Costs and Cost Analyses” OR “Costs, Cost Analysis” OR “Pricing” OR “Health Economics” OR “Health Evaluation” OR “Economic Assessment in Health” OR “Economic Assessment in Health Care” OR “Economic Assessment in Healthcare” OR “Economic Evaluation in Health” OR “Economic Evaluation in Health Care” OR “Economic Evaluation in Healthcare” OR “Analyses, Cost-Benefit” OR “Analysis, Cost-Benefit” OR “Cost-Benefit Analyses” OR “Analyses, Cost Benefit” OR “Analysis, Cost Benefit” OR “Cost Benefit Analyses” OR “Effectiveness, Cost” OR “Cost Benefit Data” OR “Data, Cost-Benefit” OR “Analyses, Cost-Utility” OR “Analysis, Cost-Utility” OR “Cost Utility Analysis” OR “Cost-Utility Analyses” OR “Economic Evaluation” OR “Economic Evaluations” OR “Evaluation, Economic” OR “Evaluations, Economic” OR “Analyses, Marginal” OR “Analysis, Marginal” OR “Marginal Analyses” OR “Analysis, Cost-Effectiveness” OR “Cost Effectiveness Analysis” OR “Cost, Cost Analysis” OR “Analyses, Cost” OR “Cost Analyses” OR “Comparison, Cost” OR “Comparisons, Cost” OR “Cost Comparisons” OR “Affordabilities” OR “Analyses, Cost-Minimization” OR “Analysis, Cost-Minimization” OR “Cost Minimization Analysis” OR “Cost-Minimization Analyses” OR “Costs” OR “Cost Measure” OR “Measure, Cost” OR “Measures, Cost” OR “Health Care Costs” OR “Cost, Health Care” OR “Costs, Health Care” OR “Health Care Cost” OR “Health Costs” OR “Cost, Health” OR “Costs, Health” OR “Health Cost” OR “Healthcare Costs” OR “Cost, Healthcare” OR “Costs, Healthcare” OR “Healthcare Cost” OR “Medical Care Costs” OR “Costs, Medical Care” OR “Cost, Medical Care” OR “Medical Care Cost” OR “Treatment Costs” OR “Cost, Treatment” OR “Costs, Treatment” OR “Treatment Cost” OR “cost control” OR “cost of illness” OR “ cost effectiveness ratio” OR “cost benefit ratio” OR “audit, cost” OR “cost audit” OR “cost containment” OR “cost savings” OR “cost minimization” OR “cost of illness analysis” OR “economic aspects of illness” OR “cost utility”) | 152 | |
| **SCOPUS** | TITLE-ABS-KEY ( "Lung Neoplasms" OR "Cancer of Lung" OR "Cancer of the Lung" OR "cancer lung" OR "cancer pulmonary" OR "cancers lung" OR "cancers pulmonary" OR "Lung Cancer" OR "Lung Cancers" OR "Lung Neoplasm" OR "neoplasm lung" OR "neoplasm pulmonary" OR "neoplasms lung" OR "neoplasms pulmonary" OR "Pulmonary Cancer" OR "Pulmonary Cancers" OR "Pulmonary Neoplasm" OR "Pulmonary Neoplasms" OR "Adenocarcinoma of Lung" OR "Lung Adenocarcinoma" OR "carcinoma non small cell lung" OR "carcinoma non small cell lung" OR "Non-Small Cell Lung Cancer" OR "Non-Small-Cell Lung Carcinoma" OR "Nonsmall Cell Lung Cancer" ) AND TITLE-ABS-KEY ( "Radiosurgery" OR "CyberKnife Radiosurgeries" OR "CyberKnife Radiosurgery" OR "Gamma Knife Radiosurgeries" OR "Gamma Knife Radiosurgery" OR "LINAC Radiosurgeries" OR "LINAC Radiosurgery" OR "Linear Accelerator Radiosurgeries" OR "Linear Accelerator Radiosurgery" OR "Radiation Therapy, Stereotactic" OR "Radiation, Stereotactic" OR "Radiosurgery, CyberKnife" OR "Radiosurgery, Gamma Knife" OR "Radiosurgery, LINAC" OR "Radiosurgery, Linear Accelerator" OR "Radiosurgery, Stereotactic" OR "Radiotherapy, Stereotactic Body" OR "Stereotactic Body Radiotherapies" OR "Stereotactic Body Radiotherapy" OR "Stereotactic Radiation" OR "Stereotactic Radiation Therapies" OR "Stereotactic Radiation Therapy" OR "Stereotactic Radiations" OR "Stereotactic Radiosurgeries" OR "Stereotactic Radiosurgery" OR "Therapy, Stereotactic Radiation" OR "radiofrequency ablation" OR "stereotactic body radiation therapy" OR "radio-surgery" OR "SABR" OR "SABRT" OR "SBRT" OR "stereotactic ablative body radiation therapy" OR "stereotactic ablative body radiotherapy" OR "stereotactic ablative radiation therapy" OR "stereotactic ablative radiotherapy" OR "stereotaxic ablative radiation therapy" OR "stereotaxic ablative radiotherapy" OR "stereotaxic body radiation therapy" OR "stereotaxic body radiotherapy" OR "SRS (stereotactic radiosurgery)" OR "stereotactic radio-surgery" OR "stereotaxic radio-surgery" OR "stereotaxic radiosurgery" ) AND TITLE-ABS-KEY ( "Cost-Benefit Analysis" OR "Benefits and Costs" OR "Cost Benefit" OR "Cost Benefit Analysis" OR "Cost Effectiveness" OR "Cost-Benefit Data" OR "Cost-Effectiveness Analysis" OR "Cost-Utility Analysis" OR "Costs and Benefits" OR "Economic Evaluation" OR "Marginal Analysis" OR "Cost Efficiency Analysis" OR "Cost-Effectiveness Evaluation" OR "Costs and Cost Analysis" OR "Affordability" OR "Analysis, Cost" OR "Cost" OR "Cost Analysis" OR "Cost Comparison" OR "Cost Measures" OR "Cost-Minimization Analysis" OR "Costs and Cost Analyses" OR "Costs, Cost Analysis" OR "Pricing" OR "Health Economics" OR "Health Evaluation" OR "Economic Assessment in Health" OR "Economic Assessment in Health Care" OR "Economic Assessment in Healthcare" OR "Economic Evaluation in Health" OR "Economic Evaluation in Health Care" OR "Economic Evaluation in Healthcare" OR "Analyses, Cost-Benefit" OR "Analysis, Cost-Benefit" OR "Cost-Benefit Analyses" OR "Analyses, Cost Benefit" OR "Analysis, Cost Benefit" OR "Cost Benefit Analyses" OR "Effectiveness, Cost" OR "Cost Benefit Data" OR "Data, Cost-Benefit" OR "Analyses, Cost-Utility" OR "Analysis, Cost-Utility" OR "Cost Utility Analysis" OR "Cost-Utility Analyses" OR "Economic Evaluation" OR "Economic Evaluations" OR "Evaluation, Economic" OR "Evaluations, Economic" OR "Analyses, Marginal" OR "Analysis, Marginal" OR "Marginal Analyses" OR "Analysis, Cost-Effectiveness" OR "Cost Effectiveness Analysis" OR "Cost, Cost Analysis" OR "Analyses, Cost" OR "Cost Analyses" OR "Comparison, Cost" OR "Comparisons, Cost" OR "Cost Comparisons" OR "Affordabilities" OR "Analyses, Cost-Minimization" OR "Analysis, Cost-Minimization" OR "Cost Minimization Analysis" OR "Cost-Minimization Analyses" OR "Costs" OR "Cost Measure" OR "Measure, Cost" OR "Measures, Cost" OR "Health Care Costs" OR "Cost, Health Care" OR "Costs, Health Care" OR "Health Care Cost" OR "Health Costs" OR "Cost, Health" OR "Costs, Health" OR "Health Cost" OR "Healthcare Costs" OR "Cost, Healthcare" OR "Costs, Healthcare" OR "Healthcare Cost" OR "Medical Care Costs" OR "Costs, Medical Care" OR "Cost, Medical Care" OR "Medical Care Cost" OR "Treatment Costs" OR "Cost, Treatment" OR "Costs, Treatment" OR "Treatment Cost" OR "cost control" OR "cost of illness" OR " cost effectiveness ratio" OR "cost benefit ratio" OR "audit, cost" OR "cost audit" OR "cost containment" OR "cost savings" OR "cost minimization" OR "cost of illness analysis" OR "economic aspects of illness" OR "cost utility" ) | 209 | |
| **Web of Science** | #1 - TS=("Lung Neoplasms" OR "Cancer of Lung" OR "Cancer of the Lung" OR "cancer lung" OR "cancer pulmonary" OR "cancers lung" OR "cancers pulmonary" OR "Lung Cancer" OR "Lung Cancers" OR "Lung Neoplasm" OR "neoplasm lung" OR "neoplasm pulmonary" OR "neoplasms lung" OR "neoplasms pulmonary" OR "Pulmonary Cancer" OR "Pulmonary Cancers" OR "Pulmonary Neoplasm" OR "Pulmonary Neoplasms" OR "Adenocarcinoma of Lung" OR "Lung Adenocarcinoma" OR "carcinoma non small cell lung" OR "carcinoma non small cell lung" OR "Non-Small Cell Lung Cancer" OR "Non-Small-Cell Lung Carcinoma" OR "Nonsmall Cell Lung Cancer")  #2 - TS=(“Radiosurgery” OR “CyberKnife Radiosurgeries” OR “CyberKnife Radiosurgery” OR “Gamma Knife Radiosurgeries” OR “Gamma Knife Radiosurgery” OR “LINAC Radiosurgeries” OR “LINAC Radiosurgery” OR “Linear Accelerator Radiosurgeries” OR “Linear Accelerator Radiosurgery” OR “Radiation Therapy, Stereotactic” OR “Radiation, Stereotactic” OR “Radiosurgery, CyberKnife” OR “Radiosurgery, Gamma Knife” OR “Radiosurgery, LINAC” OR “Radiosurgery, Linear Accelerator” OR “Radiosurgery, Stereotactic” OR “Radiotherapy, Stereotactic Body” OR “Stereotactic Body Radiotherapies” OR “Stereotactic Body Radiotherapy” OR “Stereotactic Radiation” OR “Stereotactic Radiation Therapies” OR “Stereotactic Radiation Therapy” OR “Stereotactic Radiations” OR “Stereotactic Radiosurgeries” OR “Stereotactic Radiosurgery” OR “Therapy, Stereotactic Radiation” OR “radiofrequency ablation” OR “stereotactic body radiation therapy” OR “radio-surgery” OR “SABR” OR “SABRT” OR “SBRT” OR “stereotactic ablative body radiation therapy” OR “stereotactic ablative body radiotherapy” OR “stereotactic ablative radiation therapy” OR “stereotactic ablative radiotherapy” OR “stereotaxic ablative radiation therapy” OR “stereotaxic ablative radiotherapy” OR “stereotaxic body radiation therapy” OR “stereotaxic body radiotherapy” OR “SRS (stereotactic radiosurgery)” OR “stereotactic radio-surgery” OR “stereotaxic radio-surgery” OR “stereotaxic radiosurgery”)  #3 - TS=(“Cost-Benefit Analysis” OR “Benefits and Costs” OR “Cost Benefit” OR “Cost Benefit Analysis” OR “Cost Effectiveness” OR “Cost-Benefit Data” OR “Cost-Effectiveness Analysis” OR “Cost-Utility Analysis” OR “Costs and Benefits” OR “Economic Evaluation” OR “Marginal Analysis” OR “Cost Efficiency Analysis” OR “Cost-Effectiveness Evaluation” OR “Costs and Cost Analysis” OR “Affordability” OR “Analysis, Cost” OR “Cost” OR “Cost Analysis” OR “Cost Comparison” OR “Cost Measures” OR “Cost-Minimization Analysis” OR “Costs and Cost Analyses” OR “Costs, Cost Analysis” OR “Pricing” OR “Health Economics” OR “Health Evaluation” OR “Economic Assessment in Health” OR “Economic Assessment in Health Care” OR “Economic Assessment in Healthcare” OR “Economic Evaluation in Health” OR “Economic Evaluation in Health Care” OR “Economic Evaluation in Healthcare” OR “Analyses, Cost-Benefit” OR “Analysis, Cost-Benefit” OR “Cost-Benefit Analyses” OR “Analyses, Cost Benefit” OR “Analysis, Cost Benefit” OR “Cost Benefit Analyses” OR “Effectiveness, Cost” OR “Cost Benefit Data” OR “Data, Cost-Benefit” OR “Analyses, Cost-Utility” OR “Analysis, Cost-Utility” OR “Cost Utility Analysis” OR “Cost-Utility Analyses” OR “Economic Evaluation” OR “Economic Evaluations” OR “Evaluation, Economic” OR “Evaluations, Economic” OR “Analyses, Marginal” OR “Analysis, Marginal” OR “Marginal Analyses” OR “Analysis, Cost-Effectiveness” OR “Cost Effectiveness Analysis” OR “Cost, Cost Analysis” OR “Analyses, Cost” OR “Cost Analyses” OR “Comparison, Cost” OR “Comparisons, Cost” OR “Cost Comparisons” OR “Affordabilities” OR “Analyses, Cost-Minimization” OR “Analysis, Cost-Minimization” OR “Cost Minimization Analysis” OR “Cost-Minimization Analyses” OR “Costs” OR “Cost Measure” OR “Measure, Cost” OR “Measures, Cost” OR “Health Care Costs” OR “Cost, Health Care” OR “Costs, Health Care” OR “Health Care Cost” OR “Health Costs” OR “Cost, Health” OR “Costs, Health” OR “Health Cost” OR “Healthcare Costs” OR “Cost, Healthcare” OR “Costs, Healthcare” OR “Healthcare Cost” OR “Medical Care Costs” OR “Costs, Medical Care” OR “Cost, Medical Care” OR “Medical Care Cost” OR “Treatment Costs” OR “Cost, Treatment” OR “Costs, Treatment” OR “Treatment Cost” OR “cost control” OR “cost of illness” OR “ cost effectiveness ratio” OR “cost benefit ratio” OR “audit, cost” OR “cost audit” OR “cost containment” OR “cost savings” OR “cost minimization” OR “cost of illness analysis” OR “economic aspects of illness” OR “cost utility” )  #4 = #1 AND #2 AND #3 | 139 | |
| **CRD: NHS EED and HTA Databases** | (Lung Neoplasms OR Cancer of Lung OR Cancer of the Lung OR cancer lung OR cancer pulmonary OR cancers lung OR cancers pulmonary OR Lung Cancer OR Lung Cancers OR Lung Neoplasm OR neoplasm lung OR neoplasm pulmonary OR neoplasms lung OR neoplasms pulmonary OR Pulmonary Cancer OR Pulmonary Cancers OR Pulmonary Neoplasm OR Pulmonary Neoplasms OR Adenocarcinoma of Lung OR Lung Adenocarcinoma OR carcinoma non small cell lung OR carcinoma non small cell lung OR Non-Small Cell Lung Cancer OR Non-Small-Cell Lung Carcinoma OR Nonsmall Cell Lung Cancer) AND (Radiosurgery OR CyberKnife Radiosurgery OR Gamma Knife Radiosurgery OR LINAC Radiosurgery OR Linear Accelerator Radiosurgery OR radiosurgery gamma knife OR radiosurgery linear accelerator OR radiosurgery stereotactic OR Stereotactic Body Radiotherapy OR Stereotactic Radiation OR Stereotactic Radiation Therapy OR Stereotactic Radiosurgery OR Stereotactic Ablative Radiotherapy OR SABR OR SBRT) AND (Costs OR Cost Analysis OR Affordability OR analyses cost OR analyses cost minimization OR analysis cost OR analysis cost minimization OR comparison cost OR comparisons cost OR Cost OR Cost Analyses OR Cost Analysis OR Cost Comparison OR Cost Comparisons OR Cost Measure OR Cost Measures OR cost minimization analysis OR cost cost analysis OR Cost-Minimization Analyses OR cost minimization analysis OR Costs OR costs cost analysis OR measure cost OR measures cost OR Pricing OR cost benefit analysis OR analyses cost benefit OR analyses cost benefit OR analyses cost utility OR analyses marginal OR analysis cost benefit OR analysis cost benefit OR analysis cost effectiveness OR analysis cost utility OR analysis marginal OR Cost Benefit OR cost benefit analyses OR cost benefit analysis OR cost benefit data OR Cost Effectiveness OR cost effectiveness analysis OR cost utility analysis OR cost benefit analyses OR cost benefit data OR cost effectiveness analysis OR Cost-Utility Analyses OR cost utility analysis OR data cost benefit OR Economic Evaluation OR Economic Evaluations OR effectiveness cost OR evaluation economic OR evaluations economic OR Marginal Analyses OR Marginal Analysis ) | 14 | |
| **International HTA Database** | ("Lung Neoplasms" OR "Cancer of Lung" OR "Cancer of the Lung" OR "cancer lung" OR "cancer pulmonary" OR "cancers lung" OR "cancers pulmonary" OR "Lung Cancer" OR "Lung Cancers" OR "Lung Neoplasm" OR "neoplasm lung" OR "neoplasm pulmonary" OR "neoplasms lung" OR "neoplasms pulmonary" OR "Pulmonary Cancer" OR "Pulmonary Cancers" OR "Pulmonary Neoplasm" OR "Pulmonary Neoplasms" OR "Adenocarcinoma of Lung" OR "Lung Adenocarcinoma" OR "carcinoma non small cell lung" OR "carcinoma non small cell lung" OR "Non-Small Cell Lung Cancer" OR "Non-Small-Cell Lung Carcinoma" OR "Nonsmall Cell Lung Cancer") AND ("Radiosurgery" OR "CyberKnife Radiosurgery" OR "Gamma Knife Radiosurgery" OR "LINAC Radiosurgery" OR "Linear Accelerator Radiosurgery" OR "radiosurgery gamma knife" OR "radiosurgery linear accelerator" OR "radiosurgery stereotactic" OR "Stereotactic Body Radiotherapy" OR "Stereotactic Radiation" OR "Stereotactic Radiation Therapy" OR "Stereotactic Radiosurgery" OR "Stereotactic Ablative Radiotherapy" OR "SABR" OR "SBRT") | 9 | |
| **CEA Registry** | Filter Disease: Trachea, bronchus, and lung cancers  Filter Intervention: (Cancer treatment by surgery, chemotherapy, and/or radiotherapy) | 0 | |
| **EconStor** | ("Lung Neoplasms" OR "Cancer of Lung" OR "Cancer of the Lung" OR "cancer lung" OR "cancer pulmonary" OR "cancers lung" OR "cancers pulmonary" OR "Lung Cancer" OR "Lung Cancers" OR "Lung Neoplasm" OR "neoplasm lung" OR "neoplasm pulmonary" OR "neoplasms lung" OR "neoplasms pulmonary" OR "Pulmonary Cancer" OR "Pulmonary Cancers" OR "Pulmonary Neoplasm" OR "Pulmonary Neoplasms" OR "Adenocarcinoma of Lung" OR "Lung Adenocarcinoma" OR "carcinoma non small cell lung" OR "carcinoma non small cell lung" OR "Non-Small Cell Lung Cancer" OR "Non-Small-Cell Lung Carcinoma" OR "Nonsmall Cell Lung Cancer") AND ("Radiosurgery" OR "CyberKnife Radiosurgery" OR "Gamma Knife Radiosurgery" OR "LINAC Radiosurgery" OR "Linear Accelerator Radiosurgery" OR "radiosurgery gamma knife" OR "radiosurgery linear accelerator" OR "radiosurgery stereotactic" OR "Stereotactic Body Radiotherapy" OR "Stereotactic Radiation" OR "Stereotactic Radiation Therapy" OR "Stereotactic Radiosurgery" OR "Stereotactic Ablative Radiotherapy" OR "SABR" OR "SBRT") | 0 |  |
| **EconPapers** | ("Lung Neoplasms" OR "Cancer of Lung" OR "Cancer of the Lung" OR "cancer lung" OR "cancer pulmonary" OR "cancers lung" OR "cancers pulmonary" OR "Lung Cancer" OR "Lung Cancers" OR "Lung Neoplasm" OR "neoplasm lung" OR "neoplasm pulmonary" OR "neoplasms lung" OR "neoplasms pulmonary" OR "Pulmonary Cancer" OR "Pulmonary Cancers" OR "Pulmonary Neoplasm" OR "Pulmonary Neoplasms" OR "Adenocarcinoma of Lung" OR "Lung Adenocarcinoma" OR "carcinoma non small cell lung" OR "carcinoma non small cell lung" OR "Non-Small Cell Lung Cancer" OR "Non-Small-Cell Lung Carcinoma" OR "Nonsmall Cell Lung Cancer") AND ("Radiosurgery" OR "CyberKnife Radiosurgery" OR "Gamma Knife Radiosurgery" OR "LINAC Radiosurgery" OR "Linear Accelerator Radiosurgery" OR "radiosurgery gamma knife" OR "radiosurgery linear accelerator" OR "radiosurgery stereotactic" OR "Stereotactic Body Radiotherapy" OR "Stereotactic Radiation" OR "Stereotactic Radiation Therapy" OR "Stereotactic Radiosurgery" OR "Stereotactic Ablative Radiotherapy" OR "SABR" OR "SBRT") | 6 |  |
| **EconLit** | TI / AB("Lung Neoplasms" OR "Cancer of Lung" OR "Cancer of the Lung" OR "cancer lung" OR "cancer pulmonary" OR "cancers lung" OR "cancers pulmonary" OR "Lung Cancer" OR "Lung Cancers" OR "Lung Neoplasm" OR "neoplasm lung" OR "neoplasm pulmonary" OR "neoplasms lung" OR "neoplasms pulmonary" OR "Pulmonary Cancer" OR "Pulmonary Cancers" OR "Pulmonary Neoplasm" OR "Pulmonary Neoplasms" OR "Adenocarcinoma of Lung" OR "Lung Adenocarcinoma" OR "carcinoma non small cell lung" OR "carcinoma non small cell lung" OR "Non-Small Cell Lung Cancer" OR "Non-Small-Cell Lung Carcinoma" OR "Nonsmall Cell Lung Cancer") AND ("Radiosurgery" OR "CyberKnife Radiosurgery" OR "Gamma Knife Radiosurgery" OR "LINAC Radiosurgery" OR "Linear Accelerator Radiosurgery" OR "radiosurgery gamma knife" OR "radiosurgery linear accelerator" OR "radiosurgery stereotactic" OR "Stereotactic Body Radiotherapy" OR "Stereotactic Radiation" OR "Stereotactic Radiation Therapy" OR "Stereotactic Radiosurgery" OR "Stereotactic Ablative Radiotherapy" OR "SABR" OR "SBRT") | 0 |  |
| **NICE Evidence Search** | ("Lung Neoplasms" OR "Cancer of Lung" OR "Cancer of the Lung" OR "cancer lung" OR "cancer pulmonary" OR "cancers lung" OR "cancers pulmonary" OR "Lung Cancer" OR "Lung Cancers" OR "Lung Neoplasm" OR "neoplasm lung" OR "neoplasm pulmonary" OR "neoplasms lung" OR "neoplasms pulmonary" OR "Pulmonary Cancer" OR "Pulmonary Cancers" OR "Pulmonary Neoplasm" OR "Pulmonary Neoplasms" OR "Adenocarcinoma of Lung" OR "Lung Adenocarcinoma" OR "carcinoma non small cell lung" OR "carcinoma non small cell lung" OR "Non-Small Cell Lung Cancer" OR "Non-Small-Cell Lung Carcinoma" OR "Nonsmall Cell Lung Cancer") AND ("Radiosurgery" OR "CyberKnife Radiosurgery" OR "Gamma Knife Radiosurgery" OR "LINAC Radiosurgery" OR "Linear Accelerator Radiosurgery" OR "radiosurgery gamma knife" OR "radiosurgery linear accelerator" OR "radiosurgery stereotactic" OR "Stereotactic Body Radiotherapy" OR "Stereotactic Radiation" OR "Stereotactic Radiation Therapy" OR "Stereotactic Radiosurgery" OR "Stereotactic Ablative Radiotherapy" OR "SABR" OR "SBRT")  Filters: Economic evaluations; HTA | 13 |  |
| **CADTH** | (LUNG CANCER) AND stereotactic radiotherapy  Filter: Reports | 17 |  |
| **CONITEC** | “radioterapia estereotática” OR “radioterapia estereotáxica” OR “radiocirurgia” | 0 |  |
| **TOTAL** |  | **1042** |  |

Last Search performed: June 3^rd^ 2021
