# Supplementary material for: Systematic review of economic evaluations on stereotactic ablative radiotherapy (SABR) compared to other radiotherapy techniques or surgical procedures for early-stage non-small cell lung cancer
Source: Cost Eff Resour Alloc. 2023 Jan 16;21:4. doi: 10.1186/s12962-023-00415-1 (PMC9841623; doi:10.1186/s12962-023-00415-1)
Supplement: Supplementary file 2 — Additional file 2. Excluded reports. [file 12962_2023_415_MOESM2_ESM.docx]

Additional file 2 - Excluded reports

| **Report** | **Reason** |
| --- | --- |
| CyberKnife radiosurgery: Quality of life and cost considerations (FULLER, 2007) | Partial Economic Evaluation |
| Proton Beam Therapy: Clinical and Cost-Effectiveness and Guidelines for Use \| CADTH.ca (CLARK; NKANSAH, 2008) | Only abstract available |
| Particle therapy appears to be a cost-effective treatment modality as opposed to conventional 3D radiotherapy and stereotactic body radiotherapy for inoperable stage I non-small cell lung cancer patients, but more evidence is needed (GRUTTERS et al., 2008) | Only abstract available |
| TomoTherapy, Gamma Knife, and CyberKnife therapies for patients with tumors of the lung, central nervous system, or intra-abdomen: a systematic review of clinical effectiveness and cost effectiveness (BOUDREAU; CLARK; NKANSAH, 2009) | Review |
| Cost analysis of stereotactic body radiation therapy using daily cone beam computed tomography for early stage lung cancer (OWEN et al., 2009) | Only abstract available |
| Peripheral non-small cell lung cancer treated in stereotactic conditions: A prospective medico-economic French study (CLAUDE et al., 2010) | Only abstract available |
| Is stereotactic body radiotherapy warranted in medically operable stage I NSCLC? A Markov model based decision analysis (LOUIE et al., 2010a) | Only abstract available |
| Stereotactic body radiotherapy versus surgery for medically operable stage I NSCLC: A Markov model based decision analysis (LOUIE et al., 2010b) | Only abstract available |
| Stereotactic body radiotherapy versus surgery for stage I NSCLC: A Markov model based decision analysis (RODRIGUES et al., 2010) | Only abstract available |
| Cost and effectiveness of radiofrequency ablation versus surgery for Stage I non-small cell lung cancer in the elderly: Is less more? (ALEXANDER et al., 2011) | Only abstract available |
| Stereotactic radiotherapy reduces treatment cost while improving overall survival and local control over standard fractionated radiation therapy for medically inoperable non-small-cell lung cancer (LANNI et al., 2011) | Partial Economic Evaluation |
| Stereotactic body radiotherapy versus surgery for medically operable stage I non-small-cell lung cancer: A Markov model-based decision analysis (LOUIE et al., 2011) | Effectiveness analysis |
| Cost-effectiveness analysis comparing conventional versus stereotactic body radiotherapy for surgically ineligible stage I non-small cell lung cancer (MITERA et al., 2011) | Only abstract available |
| The clinical and cost-effectiveness of radiofrequency ablation for lung cancer (MCINTOSH, 2012) | Review |
| Stereotactic ablative radiation therapy for lung tumors using helical tomotherapy: A cost-effective treatment regimen (PETERS et al., 2012a) | Only abstract available |
| Stereotactic ablative radiotherapy for lung tumors using helical tomotherapy: A cost-effective treatment regimen (PETERS et al., 2012b) | Only abstract available |
| Radiosurgery and stereotactic body radiation therapy. Efficacy, safety and efficiency in primary lung cancer and pulmonary oligometastases (UBAGO PÉREZ; MÁRQUEZ PELÁEZ, 2012) | Review |
| Cost and effectiveness of radiofrequency ablation versus limited surgical resection for stage I non-small-cell lung cancer in elderly patients: Is less more? (ALEXANDER et al., 2013) | Treatment do not include SABR |
| Stereotactic radiosurgery and stereotactic body radiation therapy cost-effectiveness results (BIJLANI et al., 2013) | Review |
| The cost of stereotactic body radiotherapy in early-stage lung cancer: A multicenter cost-calculation (LIEVENS et al., 2013) | Only abstract available |
| A cost-analysis of stereotactic radiotherapy in lung cancer (PERRIER et al., 2013) | Only abstract available |
| Cost Effectiveness Analysis of Stereotactic Body Radiation Therapy for Oligometastatic Non-Small Cell Lung Cancer: Outcomes/Health Services Research. (LESTER-COLL et al., 2014) | Only abstract available |
| Estimating the population impact of introducing stereotactic ablative radiation therapy for stage I NSCLC in Canada (LOUIE et al., 2014) | Only abstract available |
| Stereotactic body radiotherapy for lung cancer: How much does it really cost? (LIEVENS et al., 2015) | Partial Economic Evaluation |
| Patient reported outcomes following stereotactic ablative radiotherapy or surgery for stage IA non-small-cell lung cancer: Results from the ROSEL multicenter randomized trial (LOUIE et al., 2015) | Partial Economic Evaluation |
| Caro Elekta quality of life following stereotactic ablative radiotherapy for early stage lung cancer: Results from the Rosel randomized controlled trial and a systematic review (LOUIE et al., 2016) | Only abstract available |
| Linear accelerator or robotic SBRT for peripheral inoperable stage I non-small cell lung cancer: A cost-effectiveness analysis (FRELINGHUYSEN; PIGNOL; NUYTTENS, 2017) | Only abstract available |
| Cost-Effectiveness of Stereotactic Radiosurgery and Stereotactic Body Radiation Therapy: a Critical Review (LESTER-COLL; SHER, 2017) | Review |
| Promising clinical outcome with long term follow-up after body gamma knife stereotactic radiosurgery for patients with early-stage non-small cell lung cancer (LI et al., 2018) | Partial Economic Evaluation |
| Medico-economics analysis of SBRT and surgery for early stage non-small cell lung cancer (PAIX et al., 2018) | Only abstract available |
| A cost-effectiveness analysis of consolidative local therapy in oligometastatic non-squamous non-small cell lung cancer (NSCLC) (PANJE et al., 2018) | Oligometastatic NSCLC |
| Expanding access to radiotherapy for low resource countries using stereotactic body radiation therapy (CHIN; IRABOR; NGWA, 2019) | Only abstract available |
| Evidence reviews for the clinical and cost effectiveness of different radiotherapy regimens with curative intent for NSCLC: Lung cancer: diagnosis and management: Evidence review D (NATIONAL INSTITUTE FOR HEALTH AND CARE EXCELLENCE (UK), 2019) | Review |
| Easy and Fast Application to Assess the Cost-Effectiveness of Stereotactic Body Radiotherapy (SBRT) Compare to Lobectomy in Early Stage Non Small Cells Lung Cancer (NSCLC). (PAIX et al., 2019) | Only abstract available |
| Evaluating Single-Institution Costs of Consolidative Radiotherapy for Oligometastatic Non-Small Cell Lung Cancer Using Time-Driven Activity-Based Costing (PEZZI et al., 2019) | Oligometastatic NSCLC |
| Evaluating single-institution resource costs of consolidative radiotherapy for oligometastatic non-small cell lung cancer using time-driven activity-based costing (PEZZI et al., 2020) | Oligometastatic NSCLC |
| Thermal ablation versus stereotactic body radiotherapy for stage I non-small cell lung cancer: A cost-effectiveness analysis. (WU et al., 2020) | Only abstract available |
