# Supplementary material for: Systematic review of economic evaluations on stereotactic ablative radiotherapy (SABR) compared to other radiotherapy techniques or surgical procedures for early-stage non-small cell lung cancer
Source: Cost Eff Resour Alloc. 2023 Jan 16;21:4. doi: 10.1186/s12962-023-00415-1 (PMC9841623; doi:10.1186/s12962-023-00415-1)
Supplement: Supplementary file 3 — Additional file 3. Quality appraisal of the evidence of the study. [file 12962_2023_415_MOESM3_ESM.docx]

Additional file 3 – Quality appraisal of the evidence of the study

|  | **Clinical effect sizes, adverse events & complications** | | |
| --- | --- | --- | --- |
| **Study** | **Best** | **Worst** | **Comment** |
| Grutters 2010 | 5. Non-analytic studies, for example, case reports, case series | NA | NA: Experimental single-arm phase II trial |
| Sher 2011 | 5. Non-analytic studies, for example, case reports, case series | NA | NA: Experimental single-arm phase II trial NA: Meta-analysis of other treatment (chemotherapy) |
| Puri 2012 | NA | Unclear | NA: Unpublished data from a cohort of patients treated at the study center |
| Shah 2013 | 4. Case control or cohort studies | 5 - Non-analytic studies, for example, case reports, case series | NA: Experimental single-arm phase II trial NA: RCT not comparing SABR with other technique NA: Meta-analysis of other treatment (chemotherapy) |
| Louie 2014b | 5. Non-analytic studies, for example, case reports, case series | NA | NA: Experimental single-arm phase II trial |
| Paix 2018b | 1. Single RCT with direct comparison between comparator therapies, measuring final outcomes | 5 - Non-analytic studies, for example, case reports, case series | NA: Experimental single-arm phase II trial NA: RCT not comparing SABR with other technique |
| Wolff 2020 | 4 - Case control or cohort studies | 5 - Non-analytic studies, for example, case reports, case series | NA: Meta-analysis of other treatment (chemotherapy)  NA: Mathematic model of metastasis growth |
|  | **Baseline clinical data** | | |
|  | **Best** | **Worst** | **Comment** |
| Grutters 2010 | Unclear |  |  |
| Sher 2011 | Unclear |  |  |
| Puri 2012 | Unclear | Unclear |  |
| Shah 2013 | Unclear |  |  |
| Louie 2014b | Unclear |  |  |
| Paix 2018b | 4 - Old case series or analysis of reliable administrative databases. Estimates from RCTs |  |  |
| Wolff 2020 | 1 - Case series or analysis of reliable administrative databases specifically conducted for the study covering patients solely from the jurisdiction of interest |  |  |
|  | **Resource use** | | |
|  | **Best** | **Worst** | **Comment** |
| Grutters 2010 | 3 - Unsourced data from previous economic evaluation – different jurisdiction |  |  |
| Sher 2011 | Unclear |  |  |
| Puri 2012 | Unclear |  |  |
| Shah 2013 | Unclear |  |  |
| Louie 2014b | Unclear |  |  |
| Paix 2018b | 3 - Unsourced data from previous economic evaluations – same jurisdiction |  |  |
| Wolff 2020 | Unclear |  |  |
|  | **Costs** | | |
|  | **Best** | **Worst** | **Comment** |
| Grutters 2010 | 2 - Recently published cost calculations based on reliable databases or data course – same jurisdiction | Unclear | Unclear: books and official government publications |
| Sher 2011 | 3 - Unsourced data from previous economic evaluation – same jurisdiction | Unclear | Unclear: books and official government publications |
| Puri 2012 | 1 - Cost calculations based on reliable databases or data sources conducted for specific study – same jurisdiction | NA | NA: reports that do not specifically address costs |
| Shah 2013 | 1 - Cost calculations based on reliable databases or data sources conducted for specific study – same jurisdiction | 4 - Recently published cost calculations based on reliable databases or data sources – different jurisdiction | Unclear: books and official government publications |
| Louie 2014b | 1 - Cost calculations based on reliable databases or data sources conducted for specific study – same jurisdiction |  |  |
| Paix 2018b | 1 - Cost calculations based on reliable databases or data sources conducted for specific study – same jurisdiction |  |  |
| Wolff 2020 | 2 - Recently published cost calculations based on reliable databases or data course – same jurisdiction |  |  |
|  | **Utilities** | | |
|  | **Best** | **Worst** | **Comment** |
| Grutters 2010 | 2 - Indirect utility assessment from a patient sample with disease(s) of interest, using a tool not validated for the patient population |  |  |
| Sher 2011 | 3 - Direct utility assessment from a previous study from a sample either: (a) of the general population (b) with knowledge of the disease(s) of interest (c) of patients with the disease(s) of interest |  |  |
| Puri 2012 | NA |  | Used life years gained as outcome measure, therefore did not have a source for utility |
| Shah 2013 | 3 - Direct utility assessment from a previous study from a sample either: (a) of the general population (b) with knowledge of the disease(s) of interest (c) of patients with the disease(s) of interest | 4 - Unsourced utility data from previous study – method of elicitation unknown |  |
| Louie 2014b | 3 - Direct utility assessment from a previous study from a sample either: (a) of the general population (b) with knowledge of the disease(s) of interest (c) of patients with the disease(s) of interest | NA |  |
| Paix 2018b | 3 - Direct utility assessment from a previous study from a sample either: (a) of the general population (b) with knowledge of the disease(s) of interest (c) of patients with the disease(s) of interest | 4 - Unsourced utility data from previous study – method of elicitation unknown |  |
| Wolff 2020 | 2 - Indirect utility assessment from a patient sample with disease(s) of interest, using a tool not validated for the patient population | 3 -Direct utility assessment from a previous study from a sample either: (a) of the general population (b) with knowledge of the disease(s) of interest (c) of patients with the disease(s) of interest |  |
